# Enhancing antidepressant safety surveillance: comparative analysis of adverse drug reaction signals in spontaneous reporting and healthcare claims databases

**DOI:** 10.3389/fphar.2023.1291934

**Published:** 2024-01-08

**Authors:** Taehyung Kim, Xinying Jiang, Youran Noh, Maryanne Kim, Song Hee Hong

**Affiliations:** ^1^ Colleage of Pharmacy, Seoul National University, Seoul, Republic of Korea; ^2^ Research Institute of Pharmaceutical Science, College of Pharmacy, Seoul National University, Seoul, Republic of Korea; ^3^ Healthcare and Life Sciences in China and Renaissance Group, Shanghai, China

**Keywords:** signal detection, drug safety surveillance, spontaneous reporting system, healthcare claim database, antidepressant, adverse drug reaction, pharmacovigilance

## Abstract

**Background/Objective:** Spontaneous reporting systems (SRS) such as the Korea Adverse Event Reporting System (KAERS) are limited in their ability to detect adverse drug reaction (ADR) signals due to their limited data on drug use. Conversely, the national health insurance claim (NHIC) data include drug use information for all qualifying residents. This study aimed to compare ADR signal profiles for antidepressants between KAERS and NHIC, evaluating the extent to which detected signals belong to common ADRs and labeling information.

**Materials and Methods:** ADR signal detection in KAERS and NHIC databases, spanning January to December 2017, employed disproportionality analysis. Signal classes were determined based on System Organ Class (SOC) of the Medical Dictionary for Regulatory Activities (MedDRA). Also, Common ADR Coverage (CAC), the proportion of detected signals deemed common ADRs, and labeling information coverage (LIC) represented by mean average precision (mAP) were calculated. Additionally, protopathic bias and relative risk (RR) evaluation were performed to check for signal robustness.

**Results:** Signal detection revealed 51 and 62 signals in KAERS and NHIC databases, respectively. Both systems predominantly captured signals related to nervous system disorders, comprising 33.3% (N = 17) in KAERS and 50.8% (N = 31) in NHIC. Regarding the type of antidepressants, KAERS predominantly reported signals associated with tricyclic antidepressants (TCAs) (N = 21, 41.2%), while NHIC produced most signals linked to selective serotonin reuptake inhibitors (SSRIs) (N = 22, 35.5%). KAERS exhibited higher CAC (68.63% vs. 29.03%) than NHIC. LIC was also higher in KAERS than in NHIC (mAP for EB05: 1.00 vs. 0.983); i.e., NHIC identified 5 signals not documented in drug labeling information, while KAERS found none. Among the unlabeled signals, one (Duloxetine-Myelopathy) was from protopathic bias, and two (duloxetine-myelopathy and tianeptine-osteomalacia) were statistically significant in RR.

**Conclusion:** NHIC exhibited greater capability in detecting ADR signals associated with antidepressant use, encompassing unlabeled ADR signals, compared to KAERS. NHIC also demonstrated greater potential for identifying less common ADRs. Further investigation is needed for signals detected exclusively in NHIC but not covered by labeling information. This study underscores the value of integrating different sources of data, offering substantial regulatory insights and enriching the scope of pharmacovigilance.

## 1 Introduction

Pharmacovigilance relies on robust data sources to detect adverse drug reaction (ADR) signals and ensure patient safety. Spontaneous reporting systems (SRS) have traditionally been a cornerstone of pharmacovigilance, with the Korea Adverse Event Reporting System (KAERS) serving as a vital repository for adverse event reports. (van Puijenbroek et al., 2002; Van Puijenbroek et al., 2003; Bate and Evans, 2009). However, SRS, including KAERS, have a fundamental limitation—they lack comprehensive data on drug utilization, hindering their ability to detect ADR signals effectively (Hazell and Shakir, 2006).

In contrast, national health insurance claim (NHIC) databases, such as the extensive claims data from the Korean National Health Insurance Review & Assessment (HIRA) database, document records of prescription drug use for all qualifying residents in Korea (Kim et al., 2017). This presents a unique opportunity to augment traditional SRS data with information on a full set of drug exposures, potentially enhancing the detection of ADR signals.

Globally, comprehensive electronic healthcare data sources have emerged as a valuable resource for pharmacovigilance (Liu et al., 2013). In US, the Food and Drug Administration (FDA)’s Sentinel System has combined electronic health records (EHR), claims from insurance providers, pharmacy records, and patient registries from over 300 million individuals in the United States, providing a comprehensive representation of real-world healthcare practice (Carnahan et al., 2014). In Europe, the EU-ADR Project has combined electronic health records (EHR) from European countries such as UK, Italy, Denmark and Netherlands to enable large-scale drug safety monitoring (Trifiro et al., 2009; Coloma et al., 2011). While quite a many studies have utilized these databases to perform pharmacovigilance, comparing ADR signal profiles between the electronic health database to SRS is rare due to the challenges of accessing and analyzing data from multiple sources; only one study compared signal detectability between EU-ADR and FAERS (Patadia et al., 2015)

In Korea, many studies utilized the NHIC data for pharmacoepidemiologic studies (Choi et al., 2010; Kim et al., 2011a; Choi et al., 2011). However, few studies compared signal detection between NHIC and KAERS. This study aimed to compare ADR signal profiles, including signal numbers and classes for system organ class (SOC) and antidepressants between KAERS and NHIC, and to determine the extent to which detected ADR signals correspond to common ADRS and labeling information in both systems.

Given antidepressants are a widely prescribed class of medications with substantial safety issues (Uher et al., 2009), a comprehensive understanding of their ADR signals from two different data sources is essential for effective clinical decision-making. Ultimately, this research would underscore the value of combining both healthcare claims and spontaneous reporting systems, offering valuable regulatory insights.

## 2 Materials and methods

### 2.1 Data source

KAERS Data was sourced from the Korea Adverse Event Reporting System (KAERS) for the year 2017. KAERS is operated by the Korea Institute of Drug Safety and Risk Management (KIDS). We specifically selected reports containing information on the usage of antidepressants, encompassing patient demographics, drug classifications using the Anatomical Therapeutic Chemical (ATC) code, recorded adverse drug reactions (ADRs), and causality assessments based on World Health Organization-Uppsala Monitoring Centre (WHO-UMC). ADRs were cataloged following the World Health Organization-Adverse Reaction Terminology (WHO-ART).

NHIC Data are from the 2017 Health Insurance Review Agency’s National Patient Data (HIRA-NPS). HIRA-NPS is derived from a 3% random sample of the entire Korean patient population and consists of healthcare claims submitted by providers for reimbursement (Kim et al., 2011b). This dataset encompasses all prescriptions for antidepressants reported to the HIRA during the year 2017. ADRs in this dataset were identified based on patient diagnoses using the Korean Standard Classification of Diseases (KCD) codes for drug-induced disorders. The identification of antidepressants was determined through the main ingredient codes listed in each prescription. Supplementary Material provide a detailed list of identified ADRs and main ingredient codes.

**FIGURE 1:**
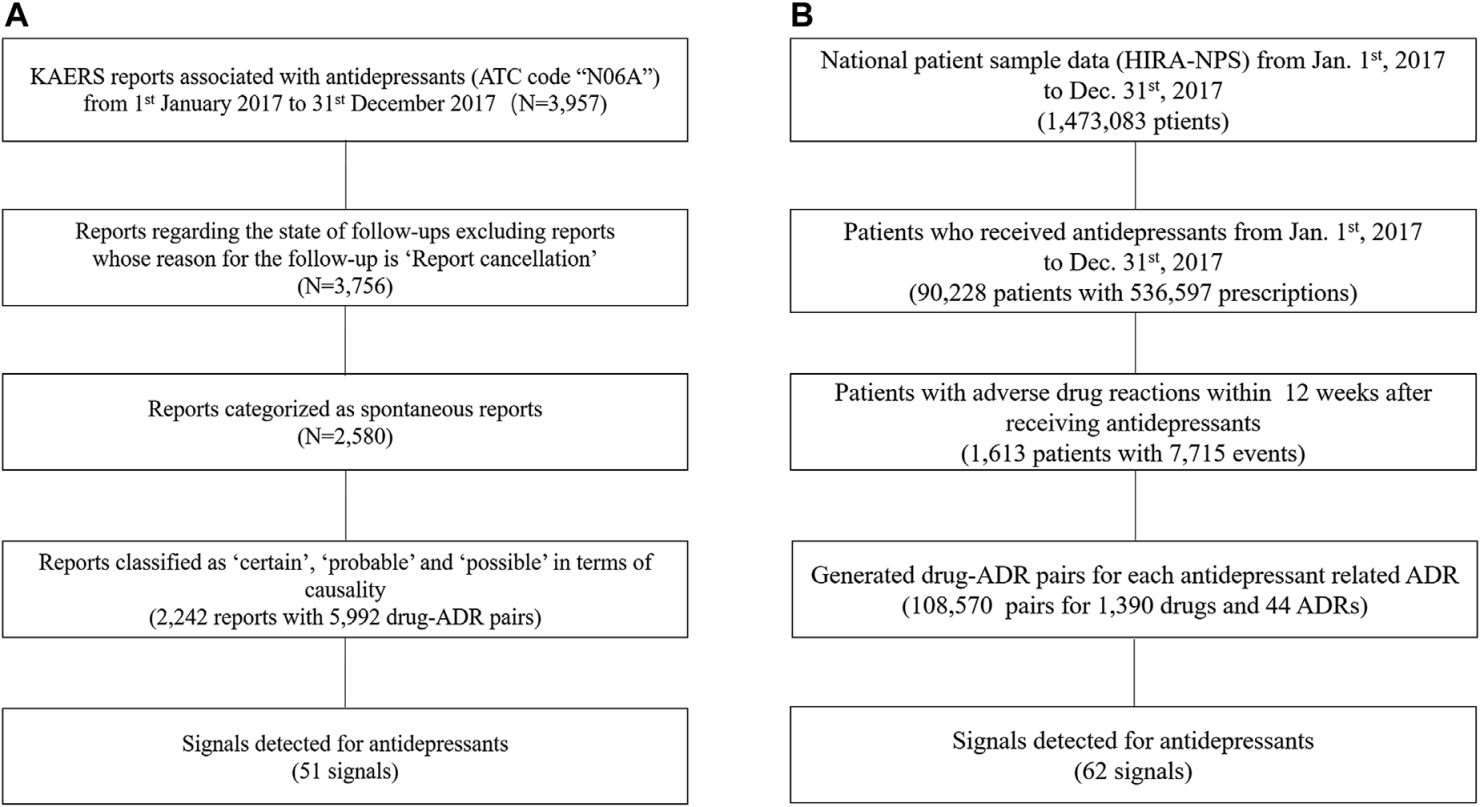
Flow Chart for Data Preparation; **(A)** KAERS data preparation; **(B)** NHIC data preparation; Abbreviation: KAERS, Korea adverse event reporting system; ATC code, Anatomical Therapeutic Chemical code; ADR, adverse drug reactions; HIRA-NPS, Health Insurance Review Agency’s National Patient Data.

In the KAERS database, we identified a total of 3,957 reports that contained antidepressants (ATC code: “N06A”) within the timeframe of 1 January 2017, to 31 December 2017 (Figure 1A). Initially, we excluded reports with the reason for follow-up listed as “report cancellation” and then selected only spontaneous reports. Among these, we retained reports classified as having “certain,” “probable,” and “possible” causality assessment based on WHO-UMC, resulting in 2,242 reports encompassing 5,992 drug-ADR pairs.

As for the NHIC database, it provided drug usage information for 1,473,083 patients during the specified time period (Figure 1B). We first identified patients who had taken antidepressants, which amounted to 90,228 patients. By narrowing down to those who had experienced a drug-induced disorder within 12 weeks after taking an antidepressant, we identified a subset of 1,613 patients. From this subset, we selected after-antidepressant ADRs and subsequently generated drug-ADR pairs, resulting in a total of 108,570 pairs involving 1,390 drugs and 44 ADRs.

**FIGURE 2:**
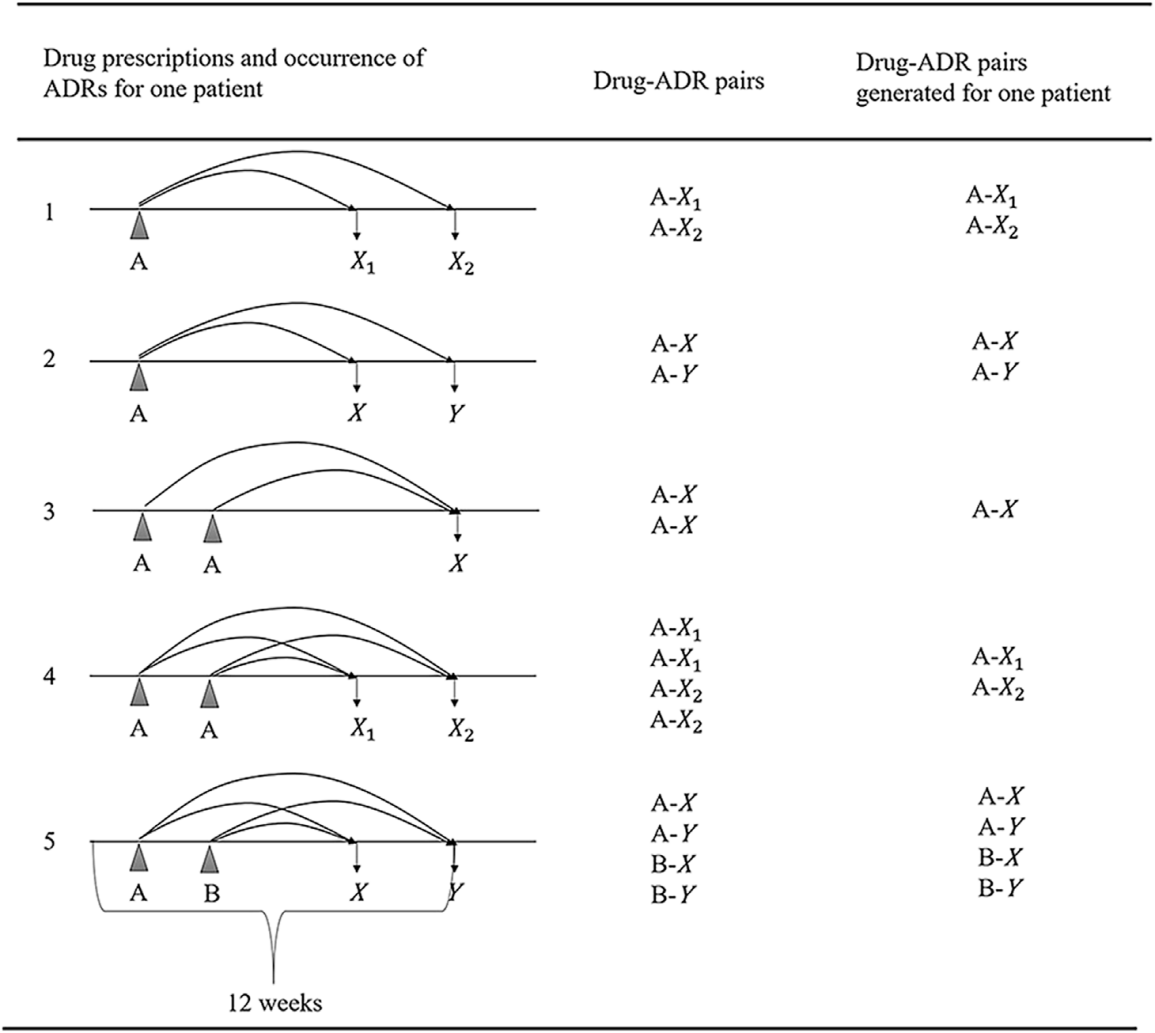
Generating algorithm for drug-ADR pairs; (A, B: Indicating different drugs; X, Y: Denoting various adverse drug reactions (ADRs); X1, X2: Indicating the same ADR occurring at different times; Abbreviation: ADR, adverse drug reaction.

Given the absence of explicit links between ADRs and drug exposure in the NHIC, we employed a systematic approach to establish these drug-ADR pairings (Figure 2).

• We initially extracted after-antidepressant ADRs, defined as ADRs occurring within 12 weeks following the last prescription of antidepressants.
• For each after-antidepressant ADR, we conducted a retrospective pairing, connecting any drugs utilized in the 12 weeks leading up to the ADR occurrence.
• Two occurrences of the same ADR (X1 and X2) within 12 weeks after taking a drug (A) were retained as A-X1 and A-X2, while different ADRs (e.g., X and Y) occurring within 12 weeks after taking different drugs (e.g., A and B) were paired as distinct drug-ADR pairs (e.g., A-X, A-Y, B-X, B-Y).
• Identical drug-ADR pairs for the same patient were considered as one to mitigate any bias arising from multiple duplications.
• We selected the 12-week time window based on established antidepressant treatment patterns and recognized practices in healthcare database studies (Choi et al., 2010; Dipiro et al., 2014).

### 2.2 Signal detection algorithms

For the detection of ADR signals, we conducted disproportionality analyses using various statistical measures, including the proportional reporting ratio (PRR), confidence interval of proportional reporting ratio (PRRCI), reporting odds ratio (ROR), and confidence interval of reporting odds ratio (RORCI). Additionally, data-mining techniques such as the information component (IC), empirical Bayesian geometric mean (EBGM), and the lower 5% point of empirical Bayesian geometric mean (EB05) were employed (Madigan et al., 2010). Table 1 presents a standard 2 × 2 contingency table for each indicator, along with the corresponding formula. The thresholds were selected in accordance with the criteria utilized in international and national SRS databases (Sciences, 2010).

**TABLE 1:** Calculation and threshold for data-mining indicators.

2 × 2 contingency table			
	Specific ADR	All other ADRs	Total
Specific drug	A (n11)	B (n12)	A + B (n10)
All other drugs	C (n21)	D (n22)	C + D (n20)
Total	A + C (n01)	B + D (n02)	A + B + C + D (n)

Corresponding formulas		
Signal detection indicators	Calculation	Thresholds
PRR	(n11/n10)/(n21/n20)	PRR≥2, $\chi^{2}$ ^(†)^≥4, n11 ≥ 3
ROR	(n11/n12)/(n21/n22)	ROR≥2, $\chi^{2}$ ≥4, n11 ≥ 3
PRRCI	(n11/n10)/(n1/n20)	PRR–1.96SE > 1, n11 ≥ 3
RORCI	(n11/n12)/(n21/n22)	ROR - 1.96SE > 1, n11 ≥ 3
IC	$\text{IC}=\log_{2}\frac{N_{ij}}{E_{ij}}$ ^(*)^	IC–2SD > 0, n11 ≥ 3
EBGM	DuMouchel (1999)	EBGM≥2.5, n11 ≥ 3
EB05	DuMouchel (1999)	EB05 ≥ 1.8, n11 ≥ 3

Abbreviations: ADR, adverse drug reaction; PRR, proportional reporting ratio; PRRCI, confidence interval of proportional reporting ratio; ROR, reporting odds ratio; RORCI, confidence interval of reporting odds ratio; IC, information component; EBGM, empirical Bayes geometric mean; EB05, the lower 5% point of empirical Bayes geometric mean; * $N_{\text{ij}}$ : observed frequency of drug-ADR pairs, $E_{\text{ij}}$ : expected frequency of drug-ADR pairs; † $\chi^{2}$ : chi-square value.

### 2.3 Comparison of detected signals

Following signal detection, all identified signals in both KAERS and NHIC were categorized and compared at the System Organ Class (SOC) level using the Medical Dictionary for Regulatory Activities (Brown et al., 1999). Additionally, signals were evaluated to determine whether they corresponded to common adverse drug reactions (ADRs) associated with antidepressants, as defined by the IBM Micromedex^®^ database, the Korea Pharmaceutical Information Center database (KPIC), as well as labeling information from the FDA in the United States and the Ministry of Food and Drug Safety (MFDS) in South Korea (IBM, 2021; KPIC, 2021). Common ADRs were considered those with an incidence rate exceeding 1% for a specific ADR related to a particular antidepressant. The proportion of common ADRs among all detected signals was calculated to assess the common ADR coverage (CAC).

The labeling information coverage (LIC) was assessed using the Mean Average Precision (mAP), a commonly used metric in information retrieval (Schuemie and safety, 2011). This metric evaluates how effectively a system ranks signals, giving higher priority to ranking true positive items. A higher mAP score signifies greater accuracy in detecting signals that align with the labeling information. It is determined by calculating the number of highly ranked signals that are true positives at the true positive point (Table 2). While there was no definitive gold standard for verifying the validity of the detected signals, the labeling information sourced from both the FDA of the United States and the MFDS of South Korea was regarded as truth in this study.

**TABLE 2:** The calculation of the mean average precision (mAP).

Drug	ADR	Indicator value	Rank by indicator	Labeling information	Precision
A	X	10	1	Yes	1/1 = 1
B	Y	9	2	No	
C	X	8	3	Yes	2/3 = 0.67
A	Z	7	4	No	
D	Y	6	5	Yes	3/5 = 0.6
mAP=(1 + 0.67+0.6)/3 = 0.76					

### 2.4 Evaluation protopathic bias and relative risk in national health insurance claim

During the signal detection process, false positive signals can emerge due to something called protopathic bias. This bias happens when a drug is prescribed to treat a disease or an early sign of a disease before that event is recorded in the database. We used a method called Longitudinal Evaluation of Observational Profiles of Adverse Events Related to Drugs (LEOPARD) to mitigate protopathic bias (Schuemie and safety, 2011). This method compares the number of prescriptions before and after a specific ADR occurs within a set time frame. If there is an increase in prescriptions after the ADR event, it suggests that the drug might be treating the ADR rather than causing it, which signifies a protopathic bias.

Additionally, we computed the Relative Risk (RR) along with its confidence interval for each drug-ADR combination to assess the robustness of the detected signals. This calculation was based on the comprehensive prescription data available in the NHIC database. Initially, number of exposures and outcomes required for the 2*2 table were organized (Table 3), and from this organized data, we computed the RR and its corresponding confidence interval. If the lower bound of the RR was greater than 1, it indicated that the risk of a particular drug causing a specific ADR was statistically significant.

**TABLE 3:** Data arrangement for relative risk calculation.

	Occurrence of specific ADR	Nonoccurrence of specific ADR	Total
Patients who took a specific antidepressant	x1	n1-x1	n1
Patients who didn’t take specific antidepressants	x2	n2-x2	n2

Abbreviations: ADR, adverse drug reaction.

All statistical analyses were conducted using SAS^®^ software (version 9.4) and R Statistical Software (version 4.0.3). Specifically, R packages such as “PhViD,” “openEBGM,” and “RCOR” were utilized for signal detection and evaluation (Sing et al., 2005; Ahmed and Poncet, 2013; Canida and Ihrie, 2017).

## 3 Results

### 3.1 Descriptive analysis of databases

In the KAERS database, the majority of reports (49.29%) originated from individuals aged 60 and above (Table 4). Of these reports, 65.7% were contributed by females, while 32.87% came from males. In terms of the types of antidepressants involved, the highest number of reports were associated with selective serotonin reuptake inhibitors (SSRIs, 39.47%), followed by tricyclic antidepressants (TCAs, 31.80%), noradrenergic and specific serotonergic antidepressants (NaSSAs, 7.00%), serotonin-norepinephrine reuptake inhibitors (SNRIs, 6.47%), serotonin antagonist and reuptake inhibitors (SARIs, 5.44%), and serotonin receptor agonists (SRAs, 3.21%).

**TABLE 4:** Description of KAERS and NHIC databases.

Characteristics	KAERS	NHIC	
	Reports associated with antidepressants	Patients receiving antidepressants	Patients with ADRs
**Total**	2,242 (100.00%)	90,228 (100.00%)	1,613 (100.00%)
Age			
0–19	52 (2.32%)	2,891 (3.20%)	33 (2.05%)
20–39	274 (12.22%)	14,182 (15.72%)	190 (11.78%)
40–59	679 (30.29%)	30,866 (34.21%)	576 (35.71%)
60+	1,105 (49.29%)	42,289 (46.87%)	814 (50.46%)
unknown	132 (5.89%)	0 (0.00%)	0 (0.00%)
Gender			
Male	737 (32.87%)	35,008 (38.80%)	656 (40.67%)
Female	1,473 (65.70%)	55,220 (61.20%)	957 (59.33%)
unknown	32 (1.43%)	0 (0.00%)	0 (0.00%)
Types of antidepressants			
SSRI	885 (39.47%)	43,596 (48.32%)^(*)^	837 (51.89%)^(*)^
TCA	713 (31.80%)	46,758 (51.82%)	751 (46.56%)
NaSSA	157 (7.00%)	4,447 (4.93%)	129 (8.00%)
SNRI	145 (6.47%)	5,140 (5.70%)	136 (8.43%)
SARI	122 (5.44%)	15,607 (17.30%)	365 (22.63%)
SRA	72 (3.21%)	1,958 (2.17%)	32 (1.98%)
other	148 (6.60%)	30 (0.03%)	0 (0.00%)
Top 5 most frequently reported ADRs in KAERS		Top 5 most after-antidepressant ADRs in NHIC	
Dizziness	362 (16.15%)	Tremor	2996 (38.83%)
Nausea	300 (13.38%)	Unspecified toxic liver disease	1276 (16.54%)
Somnolence	288 (12.85%)	Myoclonus	356 (4.61%)
Mouth dry	188 (8.39%)	Epileptic seizures	326 (4.23%)
Constipation	169 (7.54%)	Mental disorders	320 (4.15%)

Abbreviation: KAERS, korea adverse event reporting system; NHIC, national health insurance claim data; ADR, adverse drug reaction; SSRI, selective serotonin reuptake inhibitor; TCA, tricyclic antidepressant; NaSSA, noradrenergic and specific serotonergic antidepressant; SNRI, serotonin-norepinephrine reuptake inhibitor; SARI, serotonin antagonist and reuptake inhibitor; SRA, serotonin receptor agonist; (*): Percentages may total more than 100% due to multiple prescriptions for the same patient.

Similarly, in the NHIC database, the largest proportion of patients (46.87%) were aged 60 and older, and 61.20% of patients were female. The most commonly prescribed antidepressants were TCAs (51.82%) and SSRIs (48.32%), followed by SARIs (17.30%), SNRIs (5.70%), NaSSAs (4.93%), and SRAs (2.17%).

Among all the selected reports in KAERS, the most frequently reported ADRs were dizziness (16.15%), followed by nausea (13.38%), somnolence (12.85%), mouth dry (8.39%), and constipation (7.54%). Meanwhile, in NHIC, the most common after-antidepressant ADRs were tremor (38.83%), followed by unspecified toxic liver disease (16.54%), myoclonus (4.61%), epileptic seizures (4.23%), and mental disorders (4.15%).

### 3.2 Comparison detected signals between Korea adverse event reporting system and national health insurance claim

In the KAERS database, a total of 51 signals related to antidepressants were detected among 5,992 drug-ADR pairs. Notably, all of these signals corresponded to labeled adverse effects of antidepressants, as confirmed by both the FDA of the United States and the MFDS of South Korea. The antidepressant nortriptyline had the highest number of detected signals (8 signals), followed closely by amitriptyline and escitalopram (6 signals each). PRRCI generated the most signals (51 signals), closely followed by RORCI, which yielded similar results (49 signals). In contrast, EBGM and EB05 produced fewer signals, accounting for only 5 and 2 signals, respectively.

In the NHIC database, signal detection produced 62 signals. The highest number of detected signals was associated with duloxetine, which had 7 signals. Tianeptine followed with 6 signals, and amitriptyline had 5 signals. Similar to KAERS, PRRCI and RORCI were the primary indicators responsible for generating most of the signals (62 signals), while IC demonstrated similar results with 57 signals. PRR and ROR produced comparable outcomes, each resulting in 43 and 45 signals, respectively. EBGM and EB05 generated fewer signals compared to other indicators but much higher compared to KAERS, yielding only 28 and 22 signals, respectively. Out of all the detected signals, 57 were consistent with labeling information from both the FDA of the United States and the MFDS of South Korea. However, five ADRs had not yet been labeled. All the detected signal information in KAERS and HIRA is included in Supplementary Material.

**FIGURE 3:**
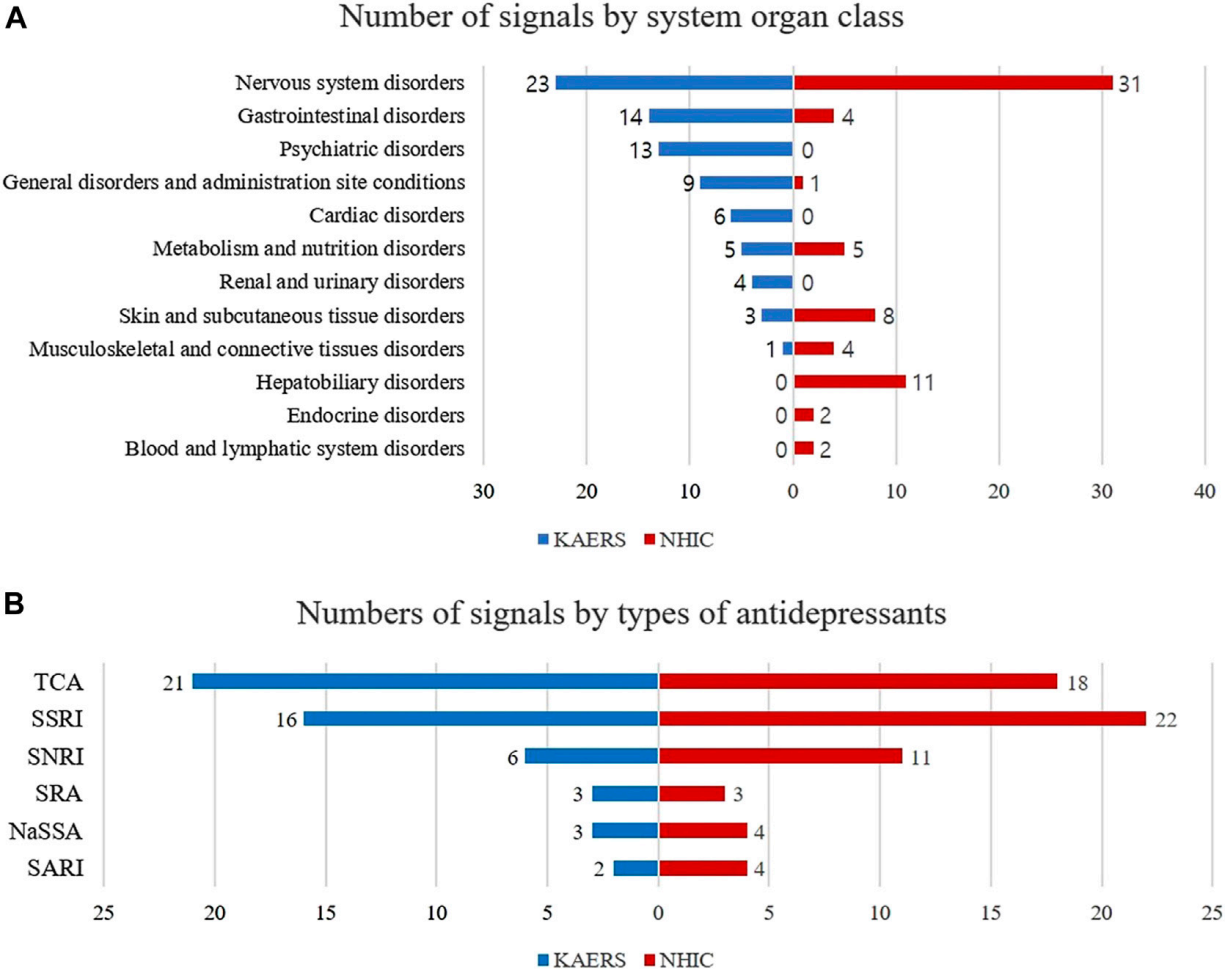
Comparison of signal profiles between KAERS and NHIC; **(A)** Number of signals by system organ class; **(B)** Number of signals by type of antidepressants; Abbreviation: KAERS, Korea adverse event reporting system; NHIC, national health insurance claim data; SSRI, selective serotonin reuptake inhibitor; TCA, tricyclic antidepressant; NaSSA, noradrenergic and specific serotonergic antidepressant; SNRI, serotonin-norepinephrine reuptake inhibitor; SARI, serotonin antagonist and reuptake inhibitor; SRA, serotonin receptor agonist.

Analyzing the profiles of the detected signals, in the KAERS database, the majority of signals were associated with nervous system disorders (N = 23, 45.1%), followed by gastrointestinal system disorders (N = 14, 27.5%), and psychiatric disorders (N = 13, 25.5%) (Figure 3). Regarding drug types, TCAs exhibited the most signals (N = 21, 41.2%), followed by SSRIs (N = 16, 31.4%), SNRIs (N = 6, 11.8%), NaSSAs (N = 3, 5.9%), SRAs (N = 3, 5.9%), and SARIs (N = 2, 3.9%).

While in NHIC database, the majority of detected signals being related to nervous system disorders (N = 31, 50.8%), followed by hepatobiliary disorders (N = 11, 18.0%), skin and appendages disorders (N = 8, 12.9%). In terms of drug types, SSRIs exhibited the most signals (N = 22, 35.5%), followed by TCAs (N = 18, 29.0%), SNRIs (N = 11, 17.7%), SARIs (N = 4, 6.5%), NaSSAs (N = 4, 6.5%), and SRAs (N = 3, 4.8%).

### 3.3 Comparison of CAC (common ADR coverage) and labeling information coverage

**FIGURE 4:**
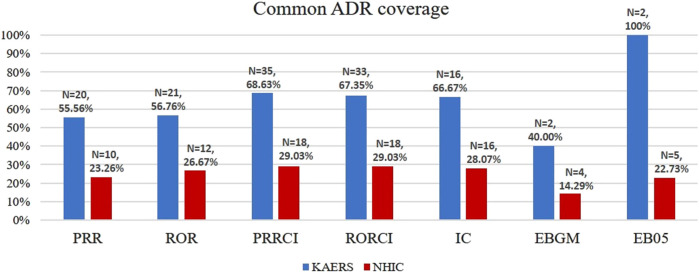
Common ADR number and common ADR coverage; Abbreviations: KAERS, Korea adverse event reporting system; NHIC, national health insurance claim data; ADR, adverse drug reaction; PRR, proportional reporting ratio; ROR, reporting odds ratio; PRRCI, confidence interval of proportional reporting ratio; RORCI, confidence interval of reporting odds ratio; IC, information component; EBGM, empirical Bayes geometric mean; EB05, the lower 5% point of empirical Bayes geometric mean.

The common ADR coverage (CAC) of detected signals was assessed in both the KAERS and NHIC systems using information from the IBM Micromedex^®^ database, the Korea Pharmaceutical Information Center database, and collected labeling information. In PRRCI, the indicator that yielded the highest number of signals in both systems, 68.63% (35 out of 51 signals) of the signals detected in KAERS were associated with common ADRs (Figure 4). In contrast, in NHIC, only 29.03% (18 out of 62 signals) of the signals were related to common ADRs.

Additionally, an assessment of labeling information coverage (LIC) was conducted to compare the two databases. In KAERS, no unlabeled signals were detected, resulting in a perfect mAP score of 1.00 for all indicators (Table 5). Conversely, in NHIC, which detected 5 unlabeled signals, EB05 exhibited the highest accuracy with a mAP of 0.983, while PRRCI and IC showed slightly lower accuracy with mAP scores of 0.936 and 0.933, respectively, according to labeling information.

**TABLE 5:** Numbers of signal detected and corresponding mAP scores.

Indicators		PRR	ROR	PRRCI	RORCI	IC	EBGM	EB05
KAERS	**Detected signals number**	36	37	51	49	24	5	2
	**Labeled signals number**	36	37	51	49	24	5	2
	**mAP**	1.00	1.00	1.00	1.00	1.00	1.00	1.00
NHIC	**Detected signals number**	43	45	62	62	57	28	22
	**Labeled signals number**	39	41	57	57	52	26	20
	**mAP**	0.944	0.955	0.936	0.947	0.933	0.951	0.983

Abbreviations: KAERS, korea adverse event reporting system; NHIC, national health insurance claim data; mAP, mean average precision; PRR, proportional reporting ratio; ROR, reporting odds ratio; PRRCI, confidence interval of proportional reporting ratio; RORCI, confidence interval of reporting odds ratio; IC, information component; EBGM, empirical Bayes geometric mean; EB05, the lower 5% point of empirical Bayes geometric mean.

### 3.4 Evaluation of signal robustness in national health insurance claim

The Longitudinal Evaluation of Observational Profiles of Adverse Events Related to Drugs (LEOPARD) method was employed to address potential protopathic bias in unlabeled detected signals (Table 6). For each unlabeled drug-ADR combination, the number of prescriptions 12 weeks before the first occurrence of the ADR and 12 weeks after the ADR were tallied along with a one-tailed binomial test. Notably, the combination of duloxetine and myelopathy is likely influenced by protopathic bias. After data processing, it was found that 34 prescriptions of duloxetine were initiated 12 weeks before the onset of myelopathy, while 44 prescriptions were created 12 weeks later. This observed significant increase (*p* < 0.05) in prescription numbers before and after the ADR strongly suggests that the signal is likely due to protopathic bias.

**TABLE 6:** LEOPARD and reporting situation for unlabeled signals.

Drug	ADR	Number of patients	Prescriptions before ADR	Prescriptions after ADR	*p*-value
Amitriptyline	Myoclonus	30	54	65	0.1797
Duloxetine	Myelopathy	13	34	44	0.01026
Tianeptine	Ulcer of oesophagus	17	24	27	0.3899
	Gastroenteritis and colitis	4	4	3	0.7734
	Osteomalacia	2	3	1	0.9375

Abbreviations: LEOPARD, longitudinal evaluation of observational profiles of adverse events related to drugs; ADR, adverse drug reaction.

In the NHIC database, for each drug-ADR combination, the Relative Risk (RR) along with its confidence interval and ADR incidence were calculated. A total of 68 combinations showed statistically significant risk compared to other antidepressants, including two of the five unlabeled signals: duloxetine-myelopathy and tianeptine-osteomalacia. A detailed list of drug-ADR combinations with a lower bound greater than 1 can be found in the Supplementary Material.

## 4 Discussion

In this study, we observed variations in the number of safety signals detected between the KAERS and NHIC databases, with 51 signals in KAERS and 62 in NHIC based on PRRCI. Notably, when we used EBGM and EB05 for signal detection, KAERS yielded a relatively smaller number of signals compared to NHIC. This discrepancy may be attributed to the significant shrinkage of the estimator in KAERS when the adverse event cell count for a specific drug is less than about 10, as reported by Madigan (Madigan, 1999).

Both KAERS and NHIC identified the majority of safety signals within the System Organ Class (SOC) of nervous system disorders, accounting for 33.3% and 50.8%, respectively. This aligns with expectations, given that most antidepressants exert their effects on neurotransmitters or their receptors, potentially leading to nervous system disorders (Khushboo and Sharma, 2017). Furthermore, antidepressants were associated with the second-highest number of safety signals in the SOC of gastrointestinal disorders (14 signals), followed by SOCs such as psychiatric disorders (13), general disorders (9), cardiac disorders (6), metabolism and nutrition disorders (5), and renal disorders (4) in KAERS. These findings are consistent with a previous meta-analysis by Correll et al., 2015, which reported increased safety risks associated with antidepressants, including obesity, dyslipidemia, diabetes mellitus, thyroid disorders, hyponatremia, and various other medical conditions.

However, NHIC exhibited a different signal profile by SOC, except for nervous system disorders. This divergence can be explained by the limitations of KCD codes used in NHIC to identify drug-induced adverse reactions. KCD codes may not adequately capture psychiatric and cardiac disorders induced by drug use, and they may also lack codes for common disorders like constipation, diarrhea, fatigue, anorexia, and dry mouth. Additionally, mild diseases such as fever, which may not prompt a clinic visit, could be missed in NHIC’s safety signal detection within the SOC of general disorders.

When we classified the signals by the types of antidepressants, KAERS showed the highest number of signals associated with TCA antidepressants (N = 21, 41.2%), followed by SSRIs (N = 16, 31.4%). In contrast, NHIC detected more signals related to SSRI antidepressants (N = 22, 35.5%) than TCAs (N = 18, 29.0%). This variation between the two databases may be influenced by differences in healthcare practices and reporting mechanisms. It's essential to note that TCAs and SSRIs are commonly prescribed classes of antidepressants, which could explain their prevalence in detected signals in both databases.

NHIC exhibited a lower Common ADR Coverage (CAC) compared to KAERS (29.03% vs. 68.63%), indicating that the safety signals detected in KAERS are more likely to consist of common adverse reactions. This substantial difference in CAC suggests that the conditions identified in NHIC based on KCD codes may not encompass many common disorders. It could also imply that NHIC has a greater potential to detect rare adverse reactions compared to KAERS. However, it could have occurred simply because the KCD codes derived from ICD codes to document medical conditions are limited in identifying drug-induced common disorders such as constipation, diarrhea, fatigue, anorexia, and dry mouth (Hohl et al., 2014).

Regarding Labeling Information Coverage (LIC), measured by the extent to which detected safety signals are mentioned in the labeling information approved by regulatory agencies, KAERS and NHIC had mAP values of 1.00 and 0.93, respectively, in EB05. NHIC notably identified 5 safety signals that were not found in the drug labeling information, including amitriptyline-myoclonus, duloxetine-myelopathy, tianeptine-ulcer of oesophagus, tianeptine-gastroenteritis and colitis, and tianeptine-osteomalacia. Among these signals, one (duloxetine-myelopathy) was attributed to protopathic bias, as duloxetine is used to treat neuropathic pain (Swidan, 2005; Hall et al., 2006). The remaining 4 signals were supported by existing literature.

For instance, the signal of amitriptyline-myoclonus was documented in a study revealing that 30 out of 98 patients who underwent cyclic antidepressant therapy experienced drug-associated myoclonus (Garvey and Tollefson, 1987). This signal was also reported in a Korean study in 2006 (Choi et al., 2006). The safety signals of ulcer of oesophagus, gastroenteritis and colitis associated with tianeptine are frequently observed in patients who have taken antidepressants (Choi et al., 2006; Kelly et al., 2008; Wang et al., 2018). The safety signal of osteomalacia, resulting from bone loss, is also documented in French and Spanish pharmacovigilance databases (Dardonville et al., 2019).

The integration of healthcare claim data with SRS data, as demonstrated in this study, offers a promising approach to enhancing the safety of antidepressant use. It enables more accurate signal detection, proactive risk management, and improved patient care, ultimately leading to safer and more effective antidepressant treatments.

### 4.1 Limitations

This study has several limitations worth noting. First, our analysis was constrained to a 12-month timeframe from the NHIC database, which necessitated limiting the KAERS data to the same 12-month period. Extending the observation period could have potentially yielded more safety signals.

Second, it's essential to acknowledge the fundamental differences in how these two systems identify safety issues. KAERS relies on voluntary and anonymous safety reports, which are associated with a higher likelihood of under-reporting and can be influenced by reporting biases driven by media coverage, financial incentives, and the duration a drug has been available on the market. In contrast, NHIC identifies drug-induced disorders based on KCD codes recorded during patients’ clinic visits, where the causality assessment between the drug and the disorders may not be as certain as in KAERS. This means that unless a patient seeks medical attention for a particular disorder and that disorder is specifically coded as drug-induced in the KCD system, it may not be captured as a safety problem in NHIC. However, prior research using NHIC for signal detection has demonstrated a relatively high positive predictive value (PPV); i.e., 80% for statin-specific adverse events, 32% for rosuvastatin-specific adverse events (Choi et al., 2010).

Lastly, while we compared the signals identified in both systems with labeling information, there is no universally accepted gold standard to definitively determine which system offers more accurate results. Therefore, direct comparisons between the two systems can pose challenges. Nevertheless, it's important to recognize that both systems provide valuable insights and play distinct roles in enhancing post-market drug surveillance efforts.

## 5 Conclusion

The NHIC exhibited greater signal detection capabilities, encompassing unlabeled ADR signals, compared to KAERS. Additionally, NHIC demonstrated a lower CAC, indicating potential for capturing more intricate signals. Further investigation is needed for signals detected exclusively in NHIC but not covered by labeling information. Integrating safety signal detection from both healthcare claims and SRS databases enhances the safety of antidepressants use and provides valuable regulatory insights for pharmacovigilance.

## Data Availability

The raw data supporting the conclusion of this article will be made available by the authors, without undue reservation.
