# Supplementary material for: Enhancing antidepressant safety surveillance: comparative analysis of adverse drug reaction signals in spontaneous reporting and healthcare claims databases
Source: Front Pharmacol. 2024 Jan 8;14:1291934. doi: 10.3389/fphar.2023.1291934 (PMC10800508; doi:10.3389/fphar.2023.1291934)
Supplement: Supplementary file 1 [file DataSheet1.docx]

Supplementary Material

Enhancing Antidepressant Safety Surveillance: Comparative Analysis of Adverse Drug Reaction Signals in Spontaneous Reporting and Healthcare Claims Databases First Author*, Co-Author, Co-Author

*** Correspondence:** Songhee Hong: songhhong@snu.ac.kr

**Supplementary Table 1. Identified ADRs in HIRA-NPS**

| **KCD** | **ADR** |
| --- | --- |
| D521 | Drug-induced folate deficiency anaemia |
| D590 | Drug-induced autoimmune haemolytic anaemia |
| D592 | Drug-induced nonautoimmune haemolytic anaemia |
| D592 | Drug-induced enzyme deficiency anaemia |
| D611 | Drug-induced aplastic anaemia |
| D642 | Secondary sideroblastic anaemia due to drugs and toxins |
| E064 | Drug-induced thyroiditis |
| F106 | Amnestic disorder, alcohol- or drug-induced |
| F116 | Amnestic disorder, opioids- or drug-induced |
| F126 | Amnestic disorder, cannabinoids- or drug-induced |
| F136 | Amnestic disorder, sedatives or hypnotics- or drug-induced |
| F146 | Amnestic disorder, cocaine- or drug-induced |
| F156 | Amnestic disorder, other stimulants, including caffeine- or drug-induced |
| F166 | Amnestic disorder, hallucinogens- or drug-induced |
| F176 | Amnestic disorder, tobacco- or drug-induced |
| F186 | Amnestic disorder, volatile solvents- or drug-induced |
| F19 | Mental and behavioural disorders due to multiple drug use and use of other psychoactive substances |
| G240 | Drug-induced dystonia |
| G251 | Drug-induced tremor |
| G253 | Drug-induced myoclonus |
| G254 | Drug-induced chorea |
| G256 | Drug-induced tics and other tics of organic origin |
| G258 | Akathisia (drug-induced) (treatment-induced) |
| G405 | Epileptic seizures related to drugs |
| G444 | Drug-induced headache, NEC |
| G620 | Drug-induced polyneuropathy |
| G711 | Drug-induced myotonia |
| G720 | Drug-induced myopathy |
| G958 | Drug-induced myelopathy |
| I427 | Cardiomyopathy due to drugs and other external agents |
| I952 | Hypotension due to drugs |
| J702 | Acute drug-induced interstitial lung disorders |
| J703 | Chronic drug-induced interstitial lung disorders |
| J704 | Drug-induced interstitial lung disorder, unspecified |
| K123 | Drug-induced mucositis (oral)(oropharyngeal) |
| K221 | Ulcer of oesophagus due to ingestion of drugs and medicaments |
| K521 | Drug-induced gastroenteritis and colitis |
| K71 | Drug-induced idiosyncratic (unpredictable) liver disease |
| K710 | Toxic liver disease |
| K711 | Toxic liver disease with cholestasis |
| K712 | Toxic liver disease with hepatic necrosis |
| K713 | Toxic liver disease with chronic persistent hepatitis |
| K714 | Toxic liver disease with chronic lobular hepatitis |
| K715 | Toxic liver disease with chronic active hepatitis |
| K716 | Toxic liver disease with hepatitis, NEC |
| K717 | Toxic liver disease with fibrosis and cirrhosis of liver |
| K718 | Toxic liver disease with other disorders of liver |
| K719 | Toxic liver disease, unspecified |
| K853 | Drug-induced acute pancreatitis |
| L105 | Drug-induced pemphigus |
| L233 | Allergic contact dermatitis due to drugs in contact with skin |
| L244 | Irritant contact dermatitis due to drugs in contact with skin |
| L251 | Unspecified contact dermatitis due to drugs in contact with skin |
| L270 | Generalized skin eruption due to drugs and medicaments |
| L271 | Localized skin eruption due to drugs and medicaments |
| L640 | Drug-induced androgenic alopecia |
| M102 | Drug-induced gout |
| M320 | Drug-induced systemic lupus erythematosus |
| M342 | Systemic sclerosis induced by drugs and chemicals |
| M804 | Drug-induced osteoporosis with patho- logical fracture |
| M814 | Drug-induced osteoporosis |
| M835 | Other drug-induced osteomalacia in adults |
| M871 | Osteonecrosis due to drugs |
| N14 | Drug-and heavy-metal- induced tubulo-interstitial and tubular condition |
| N140 | Analgesic nephropathy |
| N141 | Nephropathy induced by other drugs, medicaments and biological substances |
| N142 | Nephropathy induced by unspecified drug, medicament or biological substance |
| N144 | Toxic nephropathy, NEC |
| R502 | Drug-induced fever |
| R832 | Abnormal level of other drugs, medicaments and biological substances in cerebrospinal fluid |
| R842 | Abnormal level of other drugs, medicaments and biological substances in specimens from respiratory organs and thorax |
| R852 | Abnormal level of other drugs, medicaments and biological substances in specimens from digestive organs and abdominal |
| R862 | Abnormal level of other drugs, medicaments and biological substances in specimens from male genital organs |
| R872 | Abnormal level of other drugs, medicaments and biological substances in specimens from female genital organs |
| R892 | Abnormal level of other drugs, medicaments and biological substances in specimens from other organs, systems and tissues |
| T886 | Anaphylactic shock due to adverse effect of correct drug or medicament properly administered |
| T887 | Unspecified adverse effect of drug or medicament |

Abbreviation: KCD, Korean standard classification of diseases; ADR, adverse drug reaction; NEC, not elsewhere classified;

**Supplementary Table 2. Main ingredient codes in HIRA-NPS**

| **Drug type** | **Drug name** | **Main ingredient codes** | **Drug type** | **Drug name** | **Main ingredient codes** |
| --- | --- | --- | --- | --- | --- |
| SSRI | Fluoxetine | 161501ACH | TCA | Tianeptine | 229601ATB |
|  |  | 161501ATB |  | Imipramine | 173701ATB |
|  |  | 161502ACH |  | Clomipramine | 136301ACH |
|  |  | 161502ATB |  |  | 136302ACH |
|  | Citalopram | 428301ATB |  | Amitriptyline | 107501ATB |
|  | Paroxetine | 209301ATB |  |  | 107502ATB |
|  |  | 209302ATB |  |  | 107504ATB |
|  |  | 209304ATR |  | Doxepin | 149203ATB |
|  |  | 209305ATR |  |  | 149204ATB |
|  | Sertraline | 227001ATB |  | Amoxapine | 108002ATB |
|  |  | 227002ATB | SNRI | Venlafaxine | 247502ACR |
|  |  | 227003ATB |  |  | 247504ACR |
|  | Fluvoxamine | 162501ATB |  | Milnacipran | 355801ACH |
|  |  | 162502ATB |  |  | 355802ACH |
|  | Escitalopram | 474801ATB |  |  | 355803ACH |
|  |  | 474802ATB |  | Bupropion | 428101ATB |
|  |  | 474803ATB |  |  | 428102ATR |
|  |  | 474804ATB |  |  | 428103ATR |
|  | Duloxetine | 495501ACE | SARI | Trazodone | 242901ACH |
|  |  | 495501ATE |  |  | 242901ATB |
|  |  | 495502ACE |  |  | 242902ATB |
|  |  | 495502ATE |  |  | 242903ATR |
|  | Desvenlafaxine | 626401ATR | NaSSA | Mirtazapine | 196201ATB |
|  |  | 626402ATR |  |  | 196202ATB |
|  |  | 687601ATR |  |  | 196204ATB |
|  |  | 687602ATR | SRA | Vortioxetine | 628501ATB |
|  |  | 687701ATR |  |  | 628502ATB |
|  |  | 687702ATR |  |  | 628504ATB |

Abbreviation: SSRI, selective serotonin reuptake inhibitor; TCA, tricyclic antidepressant; NaSSA, noradrenergic and specific serotonergic antidepressant; SNRI, serotonin-norepinephrine reuptake inhibitor; SARI, serotonin antagonist and reuptake inhibitor; SRA, serotonin receptor agonist.

**Supplementary Table 3. Detected signals of antidepressants in KAERS**

| **Drug** | **ADR** | **ADR count** | **PRR** | **ROR** | **PRRCI** | **RORCI** | **IC** | **EBGM** | **EB05** |
| --- | --- | --- | --- | --- | --- | --- | --- | --- | --- |
| Amitriptyline | Somnolence | 99 | 2.71 | 3.17 | 2.23 | 2.49 | 1.23 | 2.32 | 1.94 |
|  | Dizziness | 68 | 1.29 | 1.34 | 1.03 | 1.03 | 0.33 | 1.23 | 1.01 |
|  | Mouth dry | 51 | 1.90 | 2.01 | 1.43 | 1.47 | 0.80 | 1.66 | 1.31 |
|  | Weight increase | 10 | 1.94 | 1.96 | 1.00 | 1.00 | 0.74 | 1.39 | 0.85 |
|  | Dysuria | 6 | 2.45 | 2.47 | 1.02 | 1.02 | 0.88 | 1.42 | 0.77 |
|  | Cachexia | 3 | 5.08 | 5.11 | 1.32 | 1.32 | 1.11 | 1.48 | 0.66 |
| Amoxapine | Insomnia | 5 | 13.10 | 19.82 | 6.38 | 6.57 | 2.08 | 3.61 | 1.37 |
| Bupropion | Insomnia | 7 | 3.57 | 3.85 | 1.74 | 1.74 | 1.39 | 2.09 | 1.04 |
|  | Palpitation | 4 | 4.39 | 4.58 | 1.65 | 1.63 | 1.34 | 1.78 | 0.79 |
|  | Tremor | 3 | 3.52 | 3.63 | 1.14 | 1.12 | 1.07 | 1.42 | 0.64 |
| Desvenlafaxine | Constipation | 6 | 2.44 | 2.66 | 1.15 | 1.12 | 0.98 | 1.50 | 0.80 |
|  | Anorexia | 3 | 3.56 | 3.74 | 1.18 | 1.14 | 1.08 | 1.43 | 0.65 |
| Doxepin | Headache | 3 | 3.06 | 3.30 | 1.04 | 0.99 | 0.98 | 1.35 | 0.63 |
| Duloxetine | Nausea | 129 | 2.74 | 3.14 | 2.28 | 2.52 | 1.17 | 2.22 | 1.91 |
|  | Dizziness | 103 | 1.33 | 1.38 | 1.09 | 1.10 | 0.35 | 1.25 | 1.06 |
|  | Vomiting | 68 | 2.24 | 2.37 | 1.73 | 1.79 | 0.94 | 1.85 | 1.51 |
| Escitalopram | Rash | 10 | 2.22 | 2.25 | 1.16 | 1.16 | 0.91 | 1.54 | 0.93 |
|  | Urticaria | 6 | 2.66 | 2.69 | 1.14 | 1.13 | 0.99 | 1.52 | 0.81 |
|  | Appetite increased | 5 | 4.22 | 4.27 | 1.60 | 1.60 | 1.29 | 1.82 | 0.86 |
|  | Depression | 4 | 6.45 | 6.51 | 2.06 | 2.06 | 1.43 | 1.95 | 0.83 |
|  | Hyponatraemia | 4 | 3.73 | 3.77 | 1.28 | 1.27 | 1.13 | 1.55 | 0.73 |
|  | Cachexia | 3 | 7.60 | 7.66 | 1.97 | 1.97 | 1.33 | 1.70 | 0.71 |
| Fluoxetine | Anorexia | 5 | 2.71 | 2.81 | 1.13 | 1.12 | 1.03 | 1.50 | 0.76 |
|  | Diarrhoea | 5 | 4.11 | 4.28 | 1.70 | 1.69 | 1.38 | 1.93 | 0.89 |
|  | Depression | 3 | 15.19 | 15.65 | 4.36 | 4.34 | 1.66 | 2.15 | 0.80 |
|  | Hypotension | 3 | 20.26 | 20.87 | 5.57 | 5.56 | 1.72 | 2.26 | 0.82 |
| Imipramine | Mouth dry | 5 | 3.89 | 4.80 | 1.80 | 1.75 | 1.35 | 1.91 | 0.88 |
| Milnacipran | Dyspepsia | 7 | 2.42 | 2.58 | 1.19 | 1.17 | 1.00 | 1.56 | 0.85 |
|  | Dysuria | 4 | 11.24 | 11.88 | 4.08 | 4.08 | 1.82 | 2.67 | 1.00 |
| Mirtazapine | Somnolence | 30 | 1.62 | 1.72 | 1.15 | 1.16 | 0.63 | 1.44 | 1.07 |
|  | Weight increase | 7 | 2.97 | 3.03 | 1.38 | 1.37 | 1.16 | 1.74 | 0.92 |
|  | Apathy | 6 | 4.07 | 4.16 | 1.74 | 1.74 | 1.39 | 2.03 | 0.97 |
| Nortriptyline | Mouth dry | 38 | 2.33 | 2.53 | 1.70 | 1.77 | 1.08 | 1.99 | 1.50 |
|  | Constipation | 37 | 2.49 | 2.72 | 1.81 | 1.89 | 1.16 | 2.12 | 1.58 |
|  | Palpitation | 8 | 2.29 | 2.33 | 1.11 | 1.11 | 0.92 | 1.51 | 0.86 |
|  | Temperature changed sensation | 6 | 3.48 | 3.54 | 1.48 | 1.48 | 1.23 | 1.80 | 0.90 |
|  | Micturition disorder | 5 | 4.23 | 4.29 | 1.63 | 1.63 | 1.32 | 1.85 | 0.87 |
|  | Tachycardia | 5 | 9.24 | 9.39 | 3.23 | 3.24 | 1.74 | 2.69 | 1.09 |
|  | Oliguria | 3 | 12.19 | 12.32 | 2.93 | 2.93 | 1.49 | 1.91 | 0.75 |
|  | Sleep disorder | 3 | 4.69 | 4.73 | 1.34 | 1.34 | 1.15 | 1.50 | 0.67 |
| Sertraline | Tremor | 3 | 4.17 | 4.33 | 1.35 | 1.33 | 1.18 | 1.51 | 0.67 |
| Tianeptine | Palpitation | 6 | 4.02 | 4.18 | 1.78 | 1.78 | 1.42 | 2.08 | 0.98 |
|  | Myalgia | 5 | 5.10 | 5.28 | 2.07 | 2.06 | 1.52 | 2.18 | 0.95 |
|  | Temperature changed sensation | 5 | 6.80 | 7.05 | 2.71 | 2.72 | 1.70 | 2.56 | 1.05 |
|  | Fever | 3 | 3.67 | 3.74 | 1.15 | 1.14 | 1.08 | 1.43 | 0.65 |
| Trazodone | Delirium | 3 | 4.98 | 5.06 | 1.50 | 1.49 | 1.23 | 1.57 | 0.68 |
|  | Hypotension | 3 | 11.63 | 11.82 | 3.18 | 3.17 | 1.55 | 1.98 | 0.77 |
| Venlafaxine | Asthenia | 3 | 3.22 | 3.33 | 1.04 | 1.02 | 1.01 | 1.37 | 0.63 |
| Vortioxetine | Nausea | 14 | 1.73 | 1.85 | 1.06 | 1.04 | 0.69 | 1.41 | 0.92 |
|  | Pruritus | 9 | 4.53 | 4.87 | 2.36 | 2.40 | 1.67 | 2.83 | 1.41 |
|  | Anorexia | 5 | 2.60 | 2.69 | 1.09 | 1.07 | 1.00 | 1.47 | 0.75 |

Shaded areas indicate signals that surpass the thresholds of each indicators; Abbreviation: ADR, adverse drug reaction; PRR, proportional reporting ratio; ROR, reporting odds ratio; PRRCI, confidence interval of proportional reporting ratio; RORCI, confidence interval of reporting odds ratio; IC, information component; EBGM, empirical Bayes geometric mean; EB05, the lower 5% point of empirical Bayes geometric mean.

**Supplementary Table 4. Detected signals of antidepressants in NHIC**

| Drug | ADR | Drug-ADR pairs | PRR | ROR | PRRCI | RORCI | IC | EBGM | EB05 | RR | RRlower | ADR incidence ^(*)^ |
| --- | --- | --- | --- | --- | --- | --- | --- | --- | --- | --- | --- | --- |
| Amitriptyline | Tremor | 504 | 1.52 | 1.86 | 1.42 | 1.66 | 0.59 | 1.51 | 1.40 | 0.58 | 0.42 | 0.20% |
|  | **Myoclonus** | **103** | **1.38** | **1.42** | **1.15** | **1.16** | **0.45** | **1.36** | **1.15** | **1.42** | **0.92** | **0.13%** |
|  | Toxic liver disease | 29 | 1.85 | 1.87 | 1.29 | 1.29 | 0.83 | 1.70 | 1.25 | 0.90 | 0.36 | 0.03% |
|  | Analgesic nephropathy | 16 | 8.77 | 8.87 | 5.25 | 5.28 | 2.50 | 7.51 | 4.75 | Inf | NA | 0.00% |
|  | Headache | 14 | 1.98 | 1.99 | 1.17 | 1.17 | 0.88 | 1.68 | 1.08 | 2.37 | 0.72 | 0.02% |
| Amoxapine | Other disorders of liver | 9 | 28.52 | 69.79 | 18.83 | 24.82 | 2.90 | 22.48 | 12.47 | 5.50 | 0.76 | 0.24% |
| Bupropion | Dystonia | 29 | 15.33 | 19.85 | 11.13 | 13.04 | 3.35 | 14.40 | 10.49 | 2.02 | 0.27 | 0.05% |
|  | Mental disorders | 24 | 6.46 | 7.81 | 4.51 | 4.99 | 2.39 | 6.12 | 4.20 | 3.31 | 1.03 | 0.16% |
|  | Epileptic seizures | 11 | 2.62 | 2.78 | 1.49 | 1.49 | 1.19 | 2.04 | 1.22 | 2.02 | 0.49 | 0.11% |
|  | Pancreatitis | 4 | 16.52 | 17.05 | 6.25 | 6.24 | 2.00 | 10.22 | 2.47 | 97.15 | 8.81 | 0.11% |
| Clomipramine | Tremor | 13 | 2.19 | 3.73 | 1.53 | 1.63 | 0.96 | 1.82 | 1.14 | 3.60 | 1.35 | 1.07% |
|  | Hepatitis | 6 | 5.71 | 7.37 | 2.87 | 2.90 | 1.74 | 3.65 | 1.47 | 3.34 | 0.46 | 0.27% |
| Desvenlafaxine | Tremor | 33 | 1.96 | 2.96 | 1.55 | 1.82 | 0.91 | 1.83 | 1.37 | 2.95 | 1.22 | 0.88% |
|  | Hepatic necrosis | 9 | 3.19 | 3.54 | 1.74 | 1.75 | 1.37 | 2.29 | 1.28 | 6.28 | 2.32 | 0.70% |
|  | Localized skin eruption | 8 | 7.79 | 8.74 | 4.06 | 4.16 | 2.14 | 6.32 | 2.70 | 4.91 | 0.67 | 0.18% |
| Doxepin | Generalized skin eruption | 26 | 3.17 | 3.64 | 2.24 | 2.38 | 1.54 | 2.79 | 1.97 | 1.92 | 0.84 | 0.17% |
|  | Mental disorders | 9 | 2.00 | 2.06 | 1.06 | 1.05 | 0.85 | 1.59 | 0.92 | 2.93 | 1.16 | 0.14% |
| Duloxetine | Toxic liver disease | 149 | 1.35 | 1.47 | 1.17 | 1.22 | 0.42 | 1.33 | 1.16 | 1.39 | 1.06 | 0.61% |
|  | Epileptic seizures | 56 | 2.81 | 3.00 | 2.19 | 2.28 | 1.43 | 2.62 | 2.09 | 1.12 | 0.48 | 0.06% |
|  | Polyneuropathy | 28 | 4.05 | 4.20 | 2.81 | 2.86 | 1.85 | 3.60 | 2.52 | 12.30 | 4.38 | 0.09% |
|  | **Myelopathy** | **22** | **3.56** | **3.66** | **2.35** | **2.38** | **1.66** | **3.03** | **2.06** | **8.88** | **4.05** | **0.13%** |
|  | Other disorders of liver | 20 | 1.64 | 1.67 | 1.07 | 1.07 | 0.67 | 1.50 | 1.03 | 1.74 | 0.77 | 0.07% |
|  | Chronic lobular hepatitis | 5 | 4.60 | 4.63 | 1.90 | 1.90 | 1.50 | 2.54 | 1.08 | 12.30 | 2.06 | 0.03% |
|  | Unspecified contact dermatitis | 5 | 4.69 | 4.73 | 1.94 | 1.94 | 1.52 | 2.59 | 1.10 | 8.20 | 1.16 | 0.02% |
| Escitalopram | Tremor | 390 | 1.37 | 1.57 | 1.26 | 1.38 | 0.44 | 1.36 | 1.25 | 1.67 | 1.30 | 0.43% |
|  | Irritant contact dermatitis | 41 | 1.72 | 1.75 | 1.27 | 1.28 | 0.75 | 1.63 | 1.25 | 1.08 | 0.22 | 0.01% |
|  | Folate deficiency anaemia | 20 | 5.67 | 5.76 | 3.63 | 3.65 | 2.16 | 4.97 | 3.17 | 16.21 | 1.89 | 0.02% |
|  | Cholestasis | 9 | 4.72 | 4.75 | 2.42 | 2.43 | 1.74 | 3.30 | 1.67 | Inf | NA | 0.01% |
| Fluoxetine | Tremor | 159 | 2.05 | 3.22 | 1.84 | 2.57 | 1.02 | 2.01 | 1.76 | 1.78 | 1.24 | 0.51% |
|  | Epileptic seizures | 40 | 3.85 | 4.28 | 2.88 | 3.07 | 1.83 | 3.55 | 2.66 | 1.82 | 0.77 | 0.10% |
| Fluvoxamine | Tremor | 35 | 2.05 | 3.24 | 1.63 | 2.00 | 0.97 | 1.91 | 1.44 | 1.69 | 0.63 | 0.51% |
|  | Gastroenteritis and colitis | 10 | 8.02 | 9.27 | 4.52 | 4.72 | 2.28 | 6.98 | 3.53 | 11.31 | 3.46 | 0.38% |
| Imipramine | Tremor | 403 | 2.74 | 6.88 | 2.60 | 5.75 | 1.43 | 2.70 | 2.48 | 3.20 | 2.22 | 0.88% |
|  | Epileptic seizures | 46 | 2.32 | 2.44 | 1.76 | 1.80 | 1.16 | 2.17 | 1.69 | 2.08 | 0.75 | 0.11% |
| Milnacipran | Tremor | 31 | 1.36 | 1.56 | 1.03 | 1.01 | 0.41 | 1.30 | 0.97 | 2.27 | 1.13 | 0.67% |
|  | Hepatitis | 10 | 2.49 | 2.68 | 1.39 | 1.38 | 1.12 | 1.92 | 1.13 | 3.20 | 1.01 | 0.25% |
|  | Osteoporosis | 8 | 5.02 | 5.42 | 2.59 | 2.62 | 1.78 | 3.55 | 1.67 | 9.33 | 2.81 | 0.25% |
|  | Myopathy | 4 | 4.96 | 5.15 | 1.90 | 1.88 | 1.46 | 2.46 | 0.95 | 3.11 | 0.42 | 0.08% |
| Mirtazapine | Tremor | 127 | 1.26 | 1.38 | 1.09 | 1.12 | 0.32 | 1.24 | 1.07 | 2.13 | 1.44 | 0.61% |
|  | Mental disorders | 48 | 4.02 | 4.44 | 3.08 | 3.28 | 1.90 | 3.76 | 2.89 | 6.61 | 3.44 | 0.27% |
|  | Irritant contact dermatitis | 30 | 3.57 | 3.79 | 2.53 | 2.60 | 1.71 | 3.19 | 2.29 | 6.43 | 1.30 | 0.04% |
|  | Folate deficiency anaemia | 6 | 4.66 | 4.72 | 2.09 | 2.09 | 1.60 | 2.83 | 1.26 | 3.86 | 0.45 | 0.02% |
| Paroxetine | Tremor | 174 | 1.83 | 2.58 | 1.65 | 2.10 | 0.86 | 1.80 | 1.59 | 1.36 | 0.89 | 0.40% |
|  | Chronic active hepatitis | 10 | 14.93 | 15.31 | 7.97 | 8.04 | 2.69 | 12.51 | 7.14 | 13.95 | 0.87 | 0.02% |
| Sertraline | Tremor | 175 | 2.05 | 3.23 | 1.85 | 2.60 | 1.02 | 2.01 | 1.78 | 2.34 | 1.62 | 0.65% |
|  | Mental disorders | 17 | 1.67 | 1.70 | 1.05 | 1.04 | 0.68 | 1.50 | 1.00 | 2.55 | 1.09 | 0.12% |
| Tianeptine | Toxic liver disease | 177 | 1.62 | 1.90 | 1.43 | 1.59 | 0.68 | 1.60 | 1.41 | 0.80 | 0.62 | 0.38% |
|  | **Ulcer of oesophagus** | **42** | **2.23** | **2.33** | **1.66** | **1.70** | **1.11** | **2.08** | **1.60** | **1.57** | **0.89** | **0.09%** |
|  | Hepatic necrosis | 41 | 1.65 | 1.70 | 1.23 | 1.24 | 0.69 | 1.57 | 1.21 | 1.20 | 0.76 | 0.13% |
|  | Other disorders of liver | 29 | 2.41 | 2.49 | 1.69 | 1.71 | 1.19 | 2.17 | 1.59 | 2.80 | 1.49 | 0.09% |
|  | **Gastroenteritis and colitis** | **25** | **2.32** | **2.38** | **1.57** | **1.59** | **1.13** | **2.06** | **1.47** | **0.52** | **0.18** | **0.02%** |
|  | **Osteomalacia** | **7** | **7.72** | **7.80** | **3.64** | **3.65** | **2.04** | **5.68** | **2.21** | **Inf** | **NA** | **0.01%** |
| Trazodone | Tremor | 543 | 1.57 | 1.95 | 1.47 | 1.75 | 0.64 | 1.55 | 1.45 | 1.73 | 1.32 | 0.46% |
|  | Mental disorders | 76 | 1.85 | 1.90 | 1.48 | 1.50 | 0.85 | 1.77 | 1.46 | 4.58 | 2.59 | 0.15% |
|  | Irritant contact dermatitis | 42 | 1.45 | 1.46 | 1.07 | 1.07 | 0.51 | 1.39 | 1.07 | 1.59 | 0.32 | 0.01% |
|  | Cholestasis | 6 | 2.54 | 2.55 | 1.13 | 1.13 | 1.03 | 1.71 | 0.87 | 2.39 | 0.22 | 0.01% |
| Venlafaxine | Tremor | 112 | 2.16 | 3.62 | 1.91 | 2.74 | 1.09 | 2.10 | 1.80 | 3.65 | 2.40 | 1.03% |
|  | Mental disorders | 20 | 3.23 | 3.48 | 2.13 | 2.19 | 1.54 | 2.74 | 1.84 | 4.47 | 1.77 | 0.21% |
|  | Allergic contact dermatitis | 11 | 5.55 | 5.81 | 3.11 | 3.15 | 1.99 | 4.51 | 2.30 | 3.63 | 1.11 | 0.13% |
| Vortioxetine | Tremor | 86 | 2.52 | 5.37 | 2.23 | 3.76 | 1.30 | 2.43 | 2.03 | 2.27 | 1.30 | 0.66% |
|  | Mental disorders | 13 | 3.20 | 3.44 | 1.91 | 1.94 | 1.45 | 2.51 | 1.53 | 2.00 | 0.49 | 0.10% |
|  | Unspecified contact dermatitis | 4 | 16.43 | 16.91 | 6.20 | 6.19 | 2.00 | 10.15 | 2.44 | 15.03 | 1.56 | 0.05% |

Shaded areas indicate signals that surpass the thresholds of each indicators; *ADR Incidence = number of patients who took specific antidepressants and had the occurrence of specific ADR number of patients who took specific antidepressants; Abbreviation: ADR, adverse drug reaction; PRR, proportional reporting ratio; ROR, reporting odds ratio; PRRCI, confidence interval of proportional reporting ratio; RORCI, confidence interval of reporting odds ratio; IC, information component; EBGM, empirical Bayes geometric mean; EB05, the lower 5% point of empirical Bayes geometric mean; RR, relative risk;

**Supplementary Table 5. Drug-ADR combinations with lower bonds greater than 1 in NHIC**

| **Drug** | **ADR** | **Number of patients** | **RR** | **RR lower** | **RR**  **upper** | **ADR incidence (*)** |
| --- | --- | --- | --- | --- | --- | --- |
| Amitriptyline | Nephropathy | 4 | 5.68 | 1.04 | 31.01 | 0.02% |
|  | Osteonecrosis | 8 | 3.25 | 1.18 | 8.95 | 0.03% |
| Bupropion | Acute pancreatitis | 2 | 97.15 | 8.81 | 1070.97 | 0.11% |
|  | Hypotension | 1 | 48.58 | 3.04 | 776.34 | 0.05% |
|  | Mental disorders | 3 | 3.31 | 1.03 | 10.66 | 0.16% |
|  | Tremor | 13 | 2.45 | 1.40 | 4.26 | 0.71% |
| Clomipramine | Tremor | 4 | 3.60 | 1.35 | 9.61 | 1.07% |
| Desvenlafaxine | Mental disorders | 2 | 6.98 | 1.70 | 28.70 | 0.35% |
|  | Hepatic necrosis | 4 | 6.28 | 2.32 | 17.00 | 0.70% |
|  | Mucositis | 3 | 5.68 | 1.80 | 17.90 | 0.53% |
|  | Tremor | 5 | 2.95 | 1.22 | 7.12 | 0.88% |
| Doxepin | Acute pancreatitis | 1 | 12.30 | 1.12 | 135.60 | 0.03% |
|  | Fever | 1 | 12.30 | 1.12 | 135.60 | 0.03% |
|  | Chronic persistent hepatitis | 2 | 8.20 | 1.66 | 40.61 | 0.06% |
|  | Toxic liver disease | 3 | 3.35 | 1.00 | 11.20 | 0.09% |
|  | Mental disorders | 5 | 2.93 | 1.16 | 7.40 | 0.14% |
| Duloxetine | Polyneuropathy | 9 | 12.30 | 4.38 | 34.55 | 0.09% |
|  | Chronic lobular hepatitis | 3 | 12.30 | 2.06 | 73.61 | 0.03% |
|  | Myelopathy | 13 | 8.88 | 4.05 | 19.46 | 0.13% |
|  | Unspecified contact dermatitis | 2 | 8.20 | 1.16 | 58.21 | 0.02% |
|  | Mucositis | 16 | 1.87 | 1.09 | 3.23 | 0.16% |
|  | Unspecified toxic liver disease | 60 | 1.39 | 1.06 | 1.83 | 0.61% |
| Escitalopram | Folate deficiency anaemia | 5 | 16.21 | 1.89 | 138.73 | 0.02% |
|  | Mental disorders | 17 | 1.84 | 1.01 | 3.33 | 0.08% |
|  | Tremor | 92 | 1.67 | 1.30 | 2.14 | 0.43% |
| Fluoxetine | Tremor | 32 | 1.78 | 1.24 | 2.58 | 0.51% |
| Fluvoxamine | Osteomalacia | 1 | 113.07 | 7.08 | 1806.17 | 0.13% |
|  | Unspecified contact dermatitis | 1 | 37.69 | 3.92 | 361.95 | 0.13% |
|  | Gastroenteritis and colitis | 3 | 11.31 | 3.46 | 36.97 | 0.38% |
| Imipramine | Osteoporosis | 1 | 11.95 | 1.08 | 131.79 | 0.03% |
|  | Fever | 1 | 11.95 | 1.08 | 131.79 | 0.03% |
|  | Myopathy | 4 | 4.55 | 1.56 | 13.26 | 0.11% |
|  | Tremor | 32 | 3.20 | 2.22 | 4.62 | 0.88% |
|  | Mucositis | 9 | 2.79 | 1.40 | 5.57 | 0.25% |
| Milnacipran | Osteomalacia | 1 | 74.63 | 4.67 | 1192.53 | 0.08% |
|  | Systemic lupus erythematosus | 1 | 37.32 | 3.39 | 411.26 | 0.08% |
|  | Osteoporosis | 1 | 37.32 | 3.39 | 411.26 | 0.08% |
|  | Fever | 1 | 37.32 | 3.39 | 411.26 | 0.08% |
|  | Unspecified contact dermatitis | 1 | 24.88 | 2.59 | 238.99 | 0.08% |
|  | Nephropathy | 1 | 14.93 | 1.75 | 127.67 | 0.08% |
|  | Irritant contact dermatitis | 1 | 10.66 | 1.31 | 86.59 | 0.08% |
|  | Osteoporosis | 3 | 9.33 | 2.81 | 30.94 | 0.25% |
|  | Hepatitis | 3 | 3.20 | 1.01 | 10.14 | 0.25% |
|  | Tremor | 8 | 2.27 | 1.13 | 4.58 | 0.67% |
| Mirtazapine | Mental disorders | 12 | 6.61 | 3.44 | 12.73 | 0.27% |
|  | Irritant contact dermatitis | 2 | 6.43 | 1.30 | 31.85 | 0.04% |
|  | Localized skin eruption | 5 | 3.44 | 1.33 | 8.92 | 0.11% |
|  | Osteoporosis | 4 | 3.35 | 1.16 | 9.70 | 0.09% |
|  | Epileptic seizures | 6 | 2.63 | 1.12 | 6.17 | 0.13% |
|  | Generalized skin eruption | 9 | 2.35 | 1.18 | 4.68 | 0.20% |
|  | Tremor | 27 | 2.13 | 1.44 | 3.17 | 0.61% |
| Sertraline | Osteoporosis | 4 | 3.04 | 1.05 | 8.78 | 0.08% |
|  | Mental disorders | 6 | 2.55 | 1.09 | 6.02 | 0.12% |
|  | Tremor | 32 | 2.34 | 1.62 | 3.38 | 0.65% |
| Tianeptine | Other disorders of liver | 17 | 2.80 | 1.49 | 5.23 | 0.09% |
| Trazodone | Mental disorders | 23 | 4.58 | 2.59 | 8.12 | 0.15% |
|  | Dystonia | 10 | 3.19 | 1.43 | 7.09 | 0.06% |
|  | Epileptic seizures | 20 | 3.19 | 1.81 | 5.61 | 0.13% |
|  | Allergic contact dermatitis | 13 | 2.96 | 1.48 | 5.91 | 0.08% |
|  | Tremor | 72 | 1.73 | 1.32 | 2.26 | 0.46% |
| Venlafaxine | Mental disorders | 5 | 4.47 | 1.77 | 11.29 | 0.21% |
|  | Mucositis | 8 | 3.85 | 1.86 | 7.96 | 0.34% |
|  | Tremor | 24 | 3.65 | 2.40 | 5.54 | 1.03% |
|  | Allergic contact dermatitis | 3 | 3.63 | 1.11 | 11.88 | 0.13% |
|  | Epileptic seizures | 4 | 3.26 | 1.18 | 9.06 | 0.17% |
| Vortioxetine | Unspecified contact dermatitis | 1 | 15.03 | 1.56 | 144.41 | 0.05% |
|  | Nephropathy | 1 | 9.02 | 1.05 | 77.14 | 0.05% |
|  | Tremor | 13 | 2.27 | 1.30 | 3.96 | 0.66% |

*ADR Incidence = Number of patients who took specific antidepressants and had the occurrence of specific ADR / Number of patients who took specific antidepressants. Abbreviation: ADR, adverse drug reaction; RR, relative risk.
